# Intracardiac bone cement embolism and cardiac injury: a systematic review of 116 reported cases

**DOI:** 10.1186/s13019-026-04453-7

**Published:** 2026-06-20

**Authors:** Hunbo Shim, Changseok Jeon, Seok Soo Lee, Yebon Cho, Sang Won Kim, Jin Woo Chung, JongHyun Baek

**Affiliations:** 1 Division of Cardiac Surgery, University of Rochester Medical Center, Rochester, NY USA; 2 Department of Thoracic and Cardiovascular Surgery, Incheon Sejong Hospital, Incheon, Korea; 3 Department of Thoracic and Cardiovascular Surgery, Hanyang University College of Medicine, Changwon Hanmaeum Hospital, Changwon, Korea; 4 Medical library, College of Medicine, Yeungnam University, Daegu, Korea; 5 Medical Research Center, College of Medicine, Yeungnam University, Daegu, Korea; 6 Division of Cardiovascular and Thoracic Surgery, College of Medicine, University of Kentucky, Lexington, KY USA; 7 Department of Thoracic and Cardiovascular Surgery, Yeungnam University Medical Center, Yeungnam University College of Medicine, 170 Hyeonchung-ro, Nam-gu, Daegu, 42415 Republic of Korea

**Keywords:** Bone cement, Embolism, Intracardiac, Systematic review, Cardiac surgery

## Abstract

**Background:**

Intracardiac bone cement embolism (BCE) is a rare but potentially serious complication of spinal augmentation. Clinical awareness remains limited because these events occur at the interface of spine and cardiothoracic specialties. This study synthesizes a collection of strictly intracardiac BCE cases to describe reported clinical features, injury patterns, and management strategies.

**Methods:**

A systematic search of PubMed, Embase, and Cochrane (from inception through 2025) identified 116 unique cases after a two-tier adjudication process to eliminate duplicates. Reporting quality was assessed using a modified Joanna Briggs Institute checklist.

**Results:**

The median patient age was 69 years, and 75.9% were female. Most embolic events followed vertebroplasty (67.2%) or kyphoplasty (26.7%). Chest pain (59.5%) and dyspnea (54.3%) were the most common symptoms. Diagnostic yield was high for echocardiography (96.0%) and computed tomography (CT) (93.8%) but lower for chest X-ray (63.4%). Linear cement fragments were frequently associated with perforation (46.6%), most often involving the right ventricle (70.9%). Surgical retrieval was reported in 65.5% of cases, with concomitant structural repair in 19.2%. No deaths were attributable to surgical retrieval, whereas delayed diagnosis or treatment refusal led to fatal outcomes.

**Conclusions:**

Published reports suggest that intracardiac BCE may behave as a mechanically hazardous condition with a substantial risk of perforation. The recurring patterns observed across studies highlight the importance of timely recognition and careful management. These descriptive findings may help inform clinical awareness and generate hypotheses for future investigation.

**Supplementary Information:**

The online version contains supplementary material available at https://doi.org/10.1186/s13019-026-04453-7.

## Background

Intracardiac bone cement embolism (BCE) is an exceptionally rare but potentially fatal iatrogenic complication of spinal augmentation procedures, such as vertebroplasty or kyphoplasty. Although bone cement leakage into the venous system is a well-recognized procedural risk among spine surgeons, most embolic events involve the pulmonary vasculature and are managed conservatively [1–3]. Consequently, clinical awareness regarding intracardiac BCE remains limited. Because spine surgeons infrequently encounter cardiothoracic complications and cardiothoracic surgeons rarely encounter these iatrogenic events in routine practice, a clinical blind spot has been described at the interface of these two specialties.

The potential severity of intracardiac BCE relates to the physical characteristics of the embolus. Unlike soft thromboemboli that respond to anticoagulants, intracardiac BCE comprises rigid, jagged polymethylmethacrylate (PMMA) fragments. These “intracardiac spears” have been described as causing mechanical irritation or injury to the endocardium, valvular apparatus, and ventricular walls [4]. Understanding these mechanical interactions is important for interpreting the clinical presentations and management strategies reported in the literature.

Despite its potential clinical impact, evidence regarding intracardiac BCE remains fragmented and is derived almost entirely from isolated case reports. Prior reviews have primarily focused on the incidence of cement embolism or technical aspects of spinal procedures, leaving the cardiovascular injury patterns and management approaches less clearly characterized [5, 6]. Given the rarity of this condition and the heterogeneity of reporting practices, a descriptive synthesis of published cases is needed to summarize the range of reported presentations, injury mechanisms, and management strategies.

To address this gap, we conducted a systematic review of 116 unique intracardiac BCE cases, the largest pooled cohort analyzed to date and the first to synthesize these events from a dedicated cardiothoracic surgical perspective. By consolidating individual case reports through a cardiac surgeon’s lens, this review provides a descriptive synthesis of reported injury patterns, diagnostic pathways, and management strategies. Our aim is to summarize these reported clinical features and to generate hypotheses that may inform future investigation into this rare complication.

## Methods

### Search strategy and study selection

A systematic search was conducted in PubMed, Embase, and the Cochrane Library from database inception through January 3, 2026. The search strategy was developed using controlled vocabulary (MeSH/Emtree) and extensive free-text synonyms related to bone cement, intracardiac structures, and cardiac injury mechanisms. Wildcards, truncations, and proximity operators were incorporated to maximize sensitivity.

To ensure methodological rigor, the strategy underwent a structured, role-differentiated validation process. First, two cardiothoracic surgeons (H.S., J.B.) defined the clinical concepts and operationalized the research question into three conceptual blocks derived directly from the initial search design: (1) causative material (bone cement/PMMA exposure), (2) intracardiac or major cardiac-related anatomical locations, and (3) pathology-related or embolic events. Second, a professional medical librarian (Y.C.) peer-reviewed the resulting search strategy according to the 2015 PRESS checklist, serving as the methodological approving authority [7]. After PRESS peer review, the surgeons reviewed the finalized strategy to confirm clinical relevance and ensure comprehensive capture of the full phenotypic spectrum of intracardiac BCE.

**Fig. 1:**
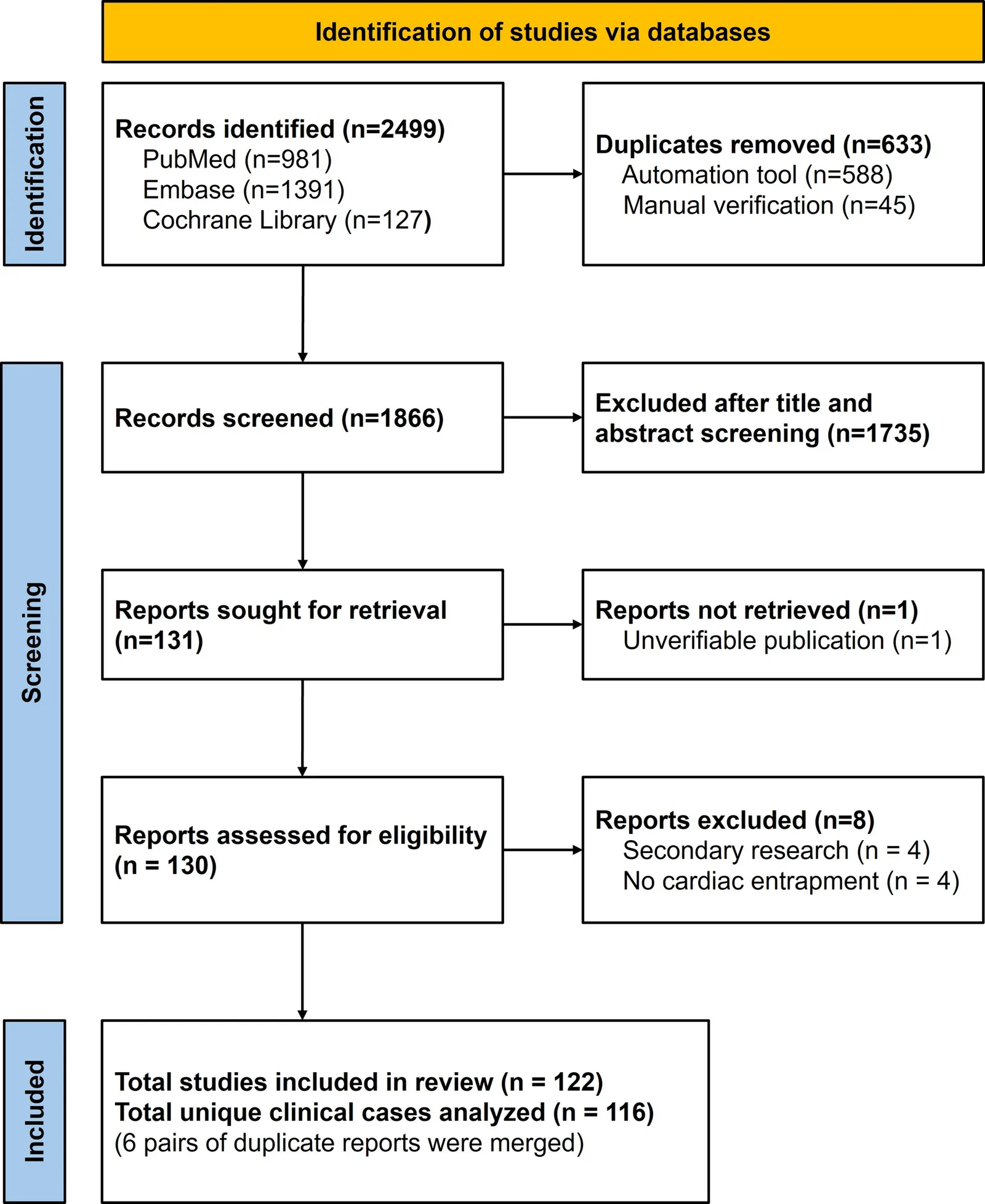
Flowchart of study identification, screening, and inclusion. The diagram illustrates the selection process for systematic review. Of the 2,499 initially identified records, 122 met the inclusion criteria. These studies were further refined into 116 unique clinical cases by merging duplicate reports that describe the same patients. Exclusion reasons at the full-text level included secondary research characteristics and the absence of confirmed intracardiac cement entrapment

This systematic review was designed and reported in accordance with Preferred Reporting Items for Systematic reviews and Meta-Analyses (PRISMA) 2020 guidelines [8]. The study selection process is illustrated in the PRISMA flow diagram (Fig. 1).

A total of 2,499 citations were retrieved (PubMed, *n* = 981; Embase, *n* = 1,391; Cochrane, *n* = 127) and imported into EndNote 21 (Clarivate, Philadelphia, PA, USA) for management. Automated deduplication with preferential retention of PubMed-indexed records yielded 1,911 records. Manual verification identified an additional 45 duplicates, resulting in 1,866 unique records for screening.

Title and abstract screening identified 131 articles for full-text review. One Embase-only citation was excluded because its publication could not be explained on the journal website. Eight full-text articles were excluded as irrelevant, leaving 122 eligible studies for detailed analysis. Full database-specific search strings are provided in Supplementary Appendix 1.

### Inclusion and exclusion criteria

We included all reports describing confirmed intracardiac bone cement embolism, identified by imaging or intraoperative findings. Reports describing isolated pulmonary cement emboli without intracardiac involvement were excluded. Conference abstracts were included to minimize publication bias in this rare condition and were appraised using the same modified JBI criteria as full‑text articles.

### Case adjudication and final cohort

To ensure the integrity of the study cohort, a rigorous and transparent approach was implemented to identify overlapping case reports—a known challenge in rare event literature. Because intracardiac BCE is highly uncommon, several clinical cases have been republished across different journals or by overlapping author groups. Rather than excluding these studies outright or inadvertently double-counting cases, we applied a structured two-tier adjudication.

Tier 1 (definitive duplication) was assigned when identical operative photographs, echocardiographic images, or radiographic figures confirmed that two publications described the same clinical event.

Tier 2 (probable duplication) referred to cases in which the convergence of rare, institution-specific clinical features made independent occurrence statistically implausible.

Consolidating six pairs of overlapping reports into single clinical cases preserved a unique, nonredundant cohort and prevented inflation of incidence estimates, aligning with best-practice principles for systematic reviews of rare case-based evidence.

### Data extraction and quality appraisal using modified joanna briggs institute checklist

Data extraction was performed independently and in parallel by three cardiothoracic surgeons (H.S., C.J., S.S.L.), who extracted all clinical, procedural, and outcome variables using a predefined data extraction template. Extracted variables included patient demographics, procedural details, cement characteristics, anatomical location of the embolus, diagnostic modalities, management strategies, and clinical outcomes.

Quality of reporting was then assessed using a modified Joanna Briggs Institute (JBI) checklist for case reports, adapted to the specific context of intracardiac bone cement embolization [9]. The checklist consisted of the following eight appraisal domains, each operationalized using predefined criteria:

(1) Demographics (age and sex explicitly reported).

(2) Timeline (explicit statement of timing or interval of the cement-related procedure).

(3) Clinical condition (symptoms described or asymptomatic status stated).

(4) Diagnostic confirmation (imaging or intraoperative modality identifying intracardiac cement).

(5) Intervention (treatment type plus ≥ 1 procedural detail; NA if explicitly no intervention).

(6) Post-intervention condition (immediate or discharge status, including death).

(7) Adverse events (explicit presence/absence).

(8) Takeaway message (at least one practice implication described).

Each domain was scored on a 0/0.5/1.0 scale based on the degree of completeness of reporting, representing incomplete, partial, and complete reporting, respectively. The detailed operational definitions for these scoring criteria are provided in Supplementary Appendix 2.

To determine the final value for each extracted variable and each JBI domain, a majority rule algorithm was applied: if two raters expressed agreement, the score was accepted. In cases of a 1–1–1 split (no majority), a structured three-rater consensus review was conducted to assign a single unified score.

### Statistical analysis

Descriptive statistics were employed to summarize the demographic, procedural, and clinical characteristics. Continuous variables, which exhibited non-normal distributions, are expressed as medians with interquartile ranges. Categorical variables are reported as absolute frequencies and percentages. Diagnostic yield was evaluated using the calculated positivity rates of initial imaging modalities.

Interrater reliability across the three raters was quantified using Fleiss’ kappa (116 cases × 8 items). Pairwise agreement analysis was also performed. Agreement strength was interpreted according to Landis and Koch’s criteria, with *k* = 0.61–0.80 indicating substantial agreement and *k* = 0.81–1.00 indicating almost-perfect agreement [10]. All analyses were conducted using R version 4.5.3 (R Foundation for Statistical Computing).

## Results

### Study selection

A total of 2,499 records were identified across PubMed, Embase, and the Cochrane Library. After automated deduplication and manual verification, 1,866 unique citations remained for screening. Of these articles, 131 underwent full-text review, and 122 met the eligibility criteria. Following a structured two-tier adjudication process to eliminate overlapping publications, 116 unique clinical cases were included in the final analysis. All frequencies reported below represent patterns among published case reports and may not reflect true clinical incidence because of inherent publication bias.

### Publication characteristics

A total of 116 cases were published between 2005 and 2025 across more than 10 countries. China (25.0%), the United States (19.8%), Korea (17.2%), and Germany (11.2%) contributed the largest proportions. Most reports were conventional case reports (54%), followed by imaging-focused reports (27%), conference abstracts (9%), letters or correspondence (7%), and small case series (3%). Publications spanned multiple disciplines, reflecting the cross-specialty nature of intracardiac BCE.

### Patient demographics and procedural characteristics

The median patient age was 69 (IQR, 61–75; valid *N* = 114), and 75.9% (88/116) were female.

Most embolic events followed vertebral augmentation procedures (113/116, 97.4%), including vertebroplasty (78/116, 67.2%), kyphoplasty (31/116, 26.7%), and cement‑augmented pedicle screw fixation (11/116, 9.5%). Three patients (2.6%) underwent non‑spinal cement procedures, all of which involved total hip replacement using cement‑augmented screws.

Among the 96 patients with documented vertebral levels, thoracic levels accounted for 82.3% (79/96), while lumbar levels involved a total of 147 segments. Reflecting multilevel treatment, this corresponds to a level-based frequency of 153.1% (147/96) based on the patient denominator. Lumbar augmentation most often involved L2 (38/96, 39.6%), L4 (33/96, 34.4%), L3 (32/96, 33.3%), and L1 (27/96, 28.1%), with L5 additionally contributing 17.7% (17/96). Simultaneous thoracolumbar cementation occurred in 27/96 (28.1%).

Most embolic episodes occurred after a first-time vertebral augmentation procedure (100/108, 92.6%; valid *N* = 108), whereas 8/108 (7.4%) had undergone prior cement-augmented procedures at different vertebral levels before the intracardiac embolism was identified.

Overall, intracardiac BCE predominantly arose during routine vertebral augmentation, particularly in the setting of multilevel cementation. Table 1 summarizes the baseline demographic and procedural characteristics of the cohort.

**Table 1:** Baseline characteristics and procedural profiles (*N* = 116)

Variable	*n*/*N*	Statistic
**Demographics**		
Age, years	−	69.0 (61.0–75.0)^*^
Female sex	88/116	75.9%
Male sex	27/116	23.3%
Unknown	1/116	0.9%
**Surgical procedure**		
Spinal procedure cohort	113/116	97.4%
Vertebroplasty	78/116	67.2%
Kyphoplasty	31/116	26.7%
Augmented screw fixation (spine)	11/116	9.5%
Non-spinal procedure (hip/pelvis)	3/116	2.6%
**Specific anatomical level of spinal procedures**	96 / 113	85.0%
Thoracic levels (T2–T12)	79/96	82.3%
T2	1/96	1.0%
T4	5/96	5.2%
T5	5/96	5.2%
T6	5/96	5.2%
T7	6/96	6.2%
T8	6/96	6.2%
T9	5/96	5.2%
T10	9/96	9.4%
T11	16/96	16.7%
T12	21/96	21.9%
Lumbar levels (L1–L5)	147/96	153.1%^†^
L1	27/96	28.1%
L2	38/96	39.6%
L3	32/96	33.3%
L4	33/96	34.4%
L5	17/96	17.7%
Sacral levels (S1)	3/96	3.1%
S1	3/96	3.1%
Simultaneous thoracolumbar	27/96	28.1%
**Procedural history**		
Primary procedure (first-time)	100/108	92.6%
Subsequent procedure (prior history)^†^	8/108	7.4%

^*^Values are expressed as n/N (%) or median (interquartile range). Calculated for patients with available age data (*n* = 114). ^†^Variables describing vertebral levels (e.g., thoracic, lumbar, L1–L5) are reported as level‑based events rather than patient‑based counts because individual patients frequently underwent procedures at multiple vertebral levels; therefore, these variables use the 96 patients with available level information as the denominator, and percentages may exceed 100%

### Clinical presentation and diagnostic performance

The median interval from the most recent cementation procedure to the definitive diagnosis was 12.5 days (IQR, 1–142.5; valid *N* = 115); although the distribution was highly skewed owing to a subset of markedly delayed presentations. Most patients were symptomatic upon detection, with only 15/116 (12.9%) remaining asymptomatic. The most frequently reported symptoms were chest pain (69/116, 59.5%) and dyspnea (63/116, 54.3%), followed by palpitation (14/116, 12.1%), hemodynamic instability (12/116, 10.3%), syncope (7/116, 6.0%), abdominal pain (4/116, 3.4%), and back pain (2/116, 1.7%). Concurrent symptoms (≥ 2) were present in 64/116 (55.2%) patients, most commonly the combination of chest pain and dyspnea. Single-symptomatic presentation accounted for 31.0% of the cohort. One patient lacked symptom documentation beyond classification as symptomatic or asymptomatic (Table 2).

**Table 2:** Clinical presentation and diagnostic timeline (*N* = 116)

Variable	*n*/*N*	Statistic (%)
**Presenting symptoms**		
Chest pain	69/116	59.5%
Dyspnea	63/116	54.3%
Palpitation	14/116	12.1%
Hemodynamic instability	12/116	10.3%
Syncope	7/116	6.0%
Abdominal pain	4/116	3.4%
Back pain	2/116	1.7%
Asymptomatic	15/116	12.9%
Unknown	1/116	0.9%
**Presentation pattern**		
Single-presenting symptom	36/116	31.0%
Multiple symptoms (≥ 2)	64/116	55.2%
**Diagnostic timeline**		
Time to diagnosis, days	−	12.5 (1.0–142.5)^*^

^*^Time‑to‑diagnosis values are expressed as median (interquartile range) and were calculated based on available data for 115 patients

Regarding diagnostic performance, chest X-ray imaging was the initial modality most frequently employed (41/116, 35.3%) but demonstrated the lowest diagnostic yield (63.4%, 26/41). In contrast, echocardiography and computed tomography (CT) exhibited high initial diagnostic yield, with 96.0% (24/25) and 93.8% (30/32), respectively. Coronary angiography/fluoroscopy, when utilized as the initial modality, also demonstrated high detection reliability (93.8%, 15/16) (Table 3). Diagnostic yield reflects the positivity rate among cases in which each modality was the first imaging test documented in the report, acknowledging that case reports do not always provide a complete description of the diagnostic sequence.

**Table 3:** Diagnostic performance of initial imaging modalities (*N* = 116)

Modality	Usage, *n*/*N* (%)	Diagnostic yield, *n*/*N* (%)
Echocardiography	25/116 (21.6%)	24/25 (96.0%)
Computed tomography	32/116 (27.6%)	30/32 (93.8%)
CAG/fluoroscopy	16/116 (13.8%)	15/16 (93.8%)
Chest X-ray	41/116 (35.3%)	26/41 (63.4%)
Others (MRI, autopsy)	2/116 (1.7%)	2/2 (100%)

Diagnostic yield refers to the positivity rate among cases in which the modality was used as the initial diagnostic test. CAG, coronary angiography

### Embolus characteristics and cardiac injury

The morphology of the intracardiac cement was predominantly linear (86/116, 74.1%), followed by curved (15/116, 12.9%) and irregular (14/116, 12.1%) configurations; one report lacked sufficient morphologic detail. Single and multiple fragments were identified in 60/116 (51.7%) and 56/116 (48.3%) cases, respectively. The most common anatomical locations were the right ventricle (RV, 87/116, 75.0%) and right atrium (73/116, 62.9%), with multi-chamber involvement observed in 48/116 (41.4%). Less frequent sites included the inferior vena cava (IVC, 4/116, 3.4%), superior vena cava (1/116, 0.9%), and left-sided chambers (left atrium, *n* = 1; left ventricle, *n* = 1; 0.9% each).

Cardiac perforation was documented in 54/116 (46.6%) patients, involving 55 discrete events, most commonly involving the RV (39 events) and right atrium (15 events), with a single event involving the aorta. Valvular involvement occurred in 18/116 (15.5%) cases, predominantly affecting the tricuspid valve (17/19 valve events). Pericardial tamponade was reported in 18/116 (15.5%) cases. These findings demonstrate that intracardiac bone cement fragments frequently penetrate adjacent cardiac structures, resulting in perforation, valvular injury, and pericardial compromise (Table 4).

**Table 4:** Embolus morphology and anatomical distribution (*N* = 116)

Category and variable	*n*/*N*	Statistic (%)
***Embolus characteristics***		
**Morphology**		
Linear	86/116	74.1%
Curved	15/116	12.9%
Irregular	14/116	12.1%
Unknown	1/116	0.9%
**Fragmentation**		
Single fragment	60/116	51.7%
Multiple fragments	56/116	48.3%
**Anatomical distribution (locations)**		
Right ventricle	87/116	75.0%
Right atrium	73/116	62.9%
Inferior vena cava	4/116	3.4%
Superior vena cava	1/116	0.9%
Left atrium	1/116	0.9%
Left ventricle	1/116	0.9%
Peritoneal cavity	1/116	0.9%
Multi-chamber involvement	48/116	41.4%
**Cardiac and vascular injuries**		
Any cardiac perforation	54/116	46.6%
Perforation sites (*n* = 55 events) ^*^		
Right ventricle	39/55	70.9%
Right atrium	15/55	27.3%
Aorta	1/55	1.8%
Valve involvement	18/116	15.5%
Valve sites (*n* = 19 events) ^†^		
Tricuspid valve	17/19	89.5%
Mitral valve	2/19	10.5%
Pericardial tamponade	18/116	15.5%

^*^Based on 55 identified perforation events among 54 patients. ^†^Based on 19 valve involvement events among 18 patients

### Management patterns

Surgical retrieval was performed in 78 patients. Of these, 76 underwent primary surgery, one required conversion after failed endovascular retrieval, and one underwent a planned hybrid procedure. Median sternotomy was the most commonly used surgical access (51/78, 65.4%), followed by thoracotomy (7/78, 9.0%), and other approaches such as robotic-assisted surgery, subxiphoid access, and laparotomy (3/78, 3.8%). The operative approach was not specified in 17/78 (21.8%). Cardiopulmonary bypass (CPB) was used in 52 of the 78 (66.7%) surgical cases, whereas 12 of the 78 (15.4%) underwent surgery without CPB support, and the remainder lacked documentation.

Concomitant cardiac procedures were performed in 15/78 (19.2%) surgical patients, including tricuspid valve surgery (*n* = 9), septal defect closure (*n* = 3), coronary revascularization (*n* = 3), mitral valve surgery (*n* = 1), and aortic repair (*n* = 1).

Endovascular retrieval was attempted in 17/116 (14.7%) patients, with a technical success rate of 76.5% (13/17). Conservative management was selected in 21/116 (18.1%) cases.

Among these, three deaths occurred in patients who declined surgical intervention despite clinical recommendations. Overall, open surgical retrieval represented the most frequently employed management strategy, whereas endovascular and conservative strategies were used in a smaller subset of patients. (Table 5).

**Table 5:** Management strategies and clinical outcomes (*N* = 116)

Category and variable	*n*/*N*	Statistic (%)
**Initial management strategy**		
Surgical intervention	76/116	65.5%
Endovascular retrieval	17/116	14.7%
Conservative management	21/116	18.1%
Autopsy-only case (no management)	1/116	0.9%
Not specified in report	1/116	0.9%
**Surgical characteristics (n = 76)**		
**Operative approach (n = 78)** ^*^		
Median sternotomy	51/78	65.4%
Thoracotomy	7/78	9.0%
Other approaches^†^	3/78	3.8%
Approach not specified	17/78	21.8%
**Use of cardiopulmonary bypass (CPB)**		
CPB-assisted surgery	52/78	66.7%
Off-pump surgery	12/78	15.4%
CPB status not specified	14/78	17.9%
**Concomitant cardiac procedures**		
Any concomitant procedure	15/78	19.2%
Procedure types (*n* = 17 events) ^‡^		
Tricuspid valve surgery	9/17	52.9%
Septal defect closure	3/17	17.6%
CABG	3/17	17.6%
Mitral valve surgery	1/17	5.9%
Aortic repair	1/17	5.9%
**Clinical outcomes**		
Uneventful discharge	95/116	81.9%
Discharged with morbidity	6/116	5.2%
Survived; discharge outcome not reported	4/116	3.4%
In-hospital mortality	8/116	6.9%
Survival status not reported	3/116	2.6%

^*^The operative approach denominator (*n* = 78) includes 76 patients who underwent primary surgical management and 2 patients who were initially managed endovascularly: one who required surgical conversion after failed retrieval and one who underwent a planned hybrid (endovascular–surgical) procedure. One case involving a small adjunctive venous access incision was classified as endovascular. ^†^Includes robotic-assisted (*n* = 1), subxiphoid (*n* = 1), and laparotomy (*n* = 1) approaches. ^‡^Based on a total of 17 concomitant procedures performed in 15 patients. ASD, atrial septal defect; CABG, coronary artery bypass grafting; CPB, cardiopulmonary bypass; PFO, patent foramen ovale

### Clinical outcomes

The overall in-hospital mortality rate was 6.9% (8/116). Survival status was reported for 113 patients, whereas 3 cases lacked documentation regarding whether the patient survived or died. Among the 105 confirmed survivors, 95 (81.9%) were discharged without reported complications and 6 (5.2%) were discharged with morbidity. The remaining 4 survivors were reported to have undergone successful treatment, but their specific discharge outcomes were not described.

Among the 6 patients with morbidity, one elderly patient managed conservatively experienced persistent symptoms attributable to retained intracardiac and intrapulmonary bone cement. The remaining five patients underwent surgical retrieval but had prolonged hospitalization, required transfer to rehabilitation facilities, or exhibited persistent cardiopulmonary dysfunction. The available reports did not allow clear attribution of these impairments to residual bone cement–related injury versus postoperative recovery.

Among the 8 in-hospital deaths, 7 occurred in patients who received conservative or endovascular management, and 1 case was identified only at autopsy without documented treatment attempts. No deaths were directly attributed to intraoperative complications of surgical retrieval.

### Reporting quality

Interrater reliability was substantial (Fleiss’ κ = 0.77). Agreement was almost perfect for demographics, clinical history or exposure, clinical presentation, diagnostic information, and management strategy (κ = 0.87–1.00). Morphological assessment demonstrated substantial reliability (κ = 0.71), and outcome reporting showed similarly strong agreement (κ = 0.80). Agreement for the takeaway message domain was lower (κ = 0.30), reflecting the inherently subjective and author-dependent nature of this item. All discrepancies were resolved using the majority rule consensus framework.

Reporting completeness based on mean consensus scores was high across all domains (overall, 93.3%): demographics, 98.7%; history/exposure, 92.7%; clinical presentation, 99.1%; diagnostic modality/timeline, 98.3%; management, 90.1%; morphology including size and shape, 87.9%; outcomes, 93.1%; and takeaway messages, 86.2%. Detailed completeness for each domain is summarized in Supplementary Appendix 3.

## Discussion

### Overview and clinical significance

This systematic review synthesizes the largest collection of strictly intracardiac BCE cases to date and represents the first dedicated analysis conducted from a cardiothoracic surgical perspective. The aggregated reports suggest that intracardiac BCE may behave as a mechanically hazardous condition rather than passive vascular obstruction [4]. High rates of cardiac perforation (46.6%) and multi-chamber involvement (41.4%) were observed among published cases. Unlike pulmonary cement emboli, which often follow a benign clinical course, these rigid and jagged “intracardiac spears” have been described as causing mechanical irritation or injury to the endocardium, valvular apparatus, and ventricular walls [2]. These observations should be interpreted within the context of publication bias, as severe or symptomatic cases are more likely to be reported.

### Publication trends and methodological quality

A gradual increase in published reports over the past two decades likely reflects the global expansion of vertebral augmentation procedures. Using a modified JBI appraisal tool, the overall methodological quality was substantial (Fleiss’ *κ* = 0.77) despite variability inherent to case-based evidence. Reporting completeness was particularly high for demographics (98.7%) and clinical presentation (99.1%), supporting the consistency of these descriptive patterns within the available literature. In contrast, the “take away message” domain showed only fair agreement (*κ* = 0.30), highlighting that although authors generally report clinical events reliably, the interpretation of their broader implications remains subjective. This variability underscores the need for more standardized, multidisciplinary reporting frameworks across spine surgery, radiology, and cardiothoracic specialties.

### Mechanical Pathway: From “Mysterious” to Visible

Historically, migration of PMMA from the vertebral venous plexus to the heart was considered a poorly understood or “mysterious” phenomenon [11]. Subsequent fluoroscopic observations by Takahashi et al. demonstrated the real-time migration of cement from the spine to the heart [12], suggesting that intracardiac BCE can occur as an immediate procedural event with a recognizable mechanical pathway. The increasing use of cement-augmented pedicle screws (9.5% of our cohort) further broadens the procedural contexts in which cement migration has been reported. These evolving observations highlight the importance of awareness during vertebral augmentation procedures, while also underscoring the need for continued investigation into the specific intraoperative factors that facilitate cement entry into the central venous system.

### Mechanical trauma and the interpretation of perforation risk

The high incidence of cardiac perforation (46.6%) and subsequent pericardial tamponade (15.5%) observed in published cases represents one of the most notable patterns in this review. Mechanically, the rigid nature of PMMA fragments may predispose to repetitive shear stress against the myocardium during each cardiac cycle, functioning as “kinetic spears” that repeatedly contact or irritate adjacent structures. However, these figures should be interpreted with caution given the inherent publication bias in case report–based evidence. Catastrophic presentations are more likely to be reported than asymptomatic or conservatively managed cases, and the 46.6% rate may therefore reflect selective reporting rather than true incidence. Even so, the consistency of these reports highlights the potential for significant mechanical injury when cement reaches the heart.

The observation that 70.9% (39/55 events) of perforations involved the right ventricle warrants a nuanced interpretation beyond simple wall thickness. In the absence of intracardiac shunts (e.g., atrial septal defect [ASD] or patent foramen ovale [PFO]), the right heart serves as the primary mechanical “trap” for emboli migrating through the systemic venous circulation. Once lodged, the RV’s complex trabeculations and its role as the most anterior and active contractile chamber create frequent high-velocity interactions between the myocardium and a rigid, stationary foreign body. This mechanical interface may help explain why perforation was most commonly reported in the RV among published cases.

### Anatomy of danger: beyond the right heart

Although most fragments lodge in the right heart, involvement of the left chambers has also been reported. Beyond migration through septal defects, such as an ASD or a PFO, spear-like fragments can directly perforate the myocardial wall to reach the left chambers [4, 13]. When this occurs, published cases describe acute valvular dysfunction or systemic hemodynamic compromise, sometimes requiring temporary mechanical support, such as intra-aortic balloon pump.

Reports by Lee et al. further illustrate that cement initially located outside the heart may later migrate into the chambers and become clinically significant [14]. Together, these observations highlight that the anatomical trajectory of cement fragments can be dynamic, and even initially stable fragments may pose risk over time.

### Diagnostic challenges and clinical mimicry

The deceptive nature of “asymptomatic” presentations in BCE is a notable pattern in the published literature. Although some cases remain stable for extended periods, delayed perforation or migration has been reported, indicating that an asymptomatic state may not reliably predict benign behavior. A striking example was described by Liu et al., in which underlying multiple myeloma masked symptoms for nearly a decade before the intracardiac fragment was incidentally detected during the COVID-19 pandemic [15]. Such cases illustrate how comorbidities, such as metastatic fractures, osteoporosis, and chronic steroid use, can create a “clinical mimicry” that misleads physicians, often leading to delays as long as 10 years.

Diagnostic challenges are further compounded by the ability of intracardiac BCE to mimic acute cardiovascular emergencies. In our cohort, this mimicry frequently resulted in delayed recognition (median interval, 12.5 days). Chest X-ray imaging has a low sensitivity (63.4%) and often misidentifies cement fragments because of the overlap with dense cardiac structures or the vertebral column, leading to confusion with surgical clips or vascular calcifications [16]. Even CT occasionally misclassified linear fragments as coronary artery or valvular calcification [16].

This radiological mimicry becomes particularly challenging when linear cement aligns with the anatomical course of the coronary arteries, such as the right coronary artery or left anterior descending artery. When combined with clinical chest pain (59.5%) and electrocardiogram changes, this pattern can prompt an initial suspicion of acute coronary syndrome [16–18]. Several reports describe patients undergoing invasive coronary angiography, with the intracardiac “spear” identified only during the procedure [19]. These presentations are likely driven by mechanical irritation of the pericardium, as fragments that penetrate or abut the myocardium can induce localized or diffuse pericarditis, explaining the associated ST-segment changes and pleuritic pain [16–18]. Collectively, these diagnostic pitfalls highlight how intracardiac BCE can closely resemble more common cardiac conditions, contributing to delays in recognition.

### Dilemma of retrieval: surgical vs endovascular approaches

The choice between endovascular and surgical retrieval remains a complex decision in BCE management, as the optimal approach depends heavily on the physical characteristics and geometry of the fragment. In our cohort, endovascular retrieval was successful in 76.5% of attempts but was also associated with secondary fragmentation in several cases. In these instances, manipulation of the fragment resulted in migration into the distal pulmonary vasculature [17, 20–23]. Although distal pulmonary emboli may be clinically tolerated, fragmentation represents a procedural complication and introduces a new embolic event.

This case-by-case review, consistent with observations by Wu et al., suggests that fragments with curved or complex geometries are particularly difficult to grasp and extract safely, even under direct surgical vision [24]. Attempting endovascular retrieval in such scenarios may increase the risk of secondary fragmentation, with smaller pieces migrating distally into the pulmonary arterial tree or creating new sites of perforation.

Delayed migration has also been reported, with fragments initially located outside the heart later entering the chambers and becoming clinically significant [14]. These observations highlight why surgical retrieval is often selected in published cases: it allows removal of the fragment in a single piece and enables direct repair of associated myocardial or valvular injury when present. In our cohort, 19.2% of surgical cases required concomitant structural repairs, such as valvuloplasty or defect closure, which cannot be addressed endovascularly.

Hybrid strategies described in the literature have shown variable rationale. For example, reports involving endovascular displacement of fragments into abdominal IVC followed by laparotomy represent a more invasive sequence than standard thoracic access [25]. The value of hybrid approaches should therefore be assessed based on whether they meaningfully reduce procedural invasiveness or risk. Many reported hybrid procedures functioned primarily as “endovascular-assisted extractions,” with definitive removal still requiring open surgery. As transcatheter technologies evolve, fully endovascular solutions may become feasible, but current evidence indicates that surgical extraction remains the most reliable method for ensuring complete removal and addressing associated structural injury when present.

### Limitations

This systematic review has several limitations. First, case report–based evidence is inherently vulnerable to publication bias as severe or symptomatic presentations are more likely to be reported than asymptomatic or conservatively managed cases. The high perforation rate observed in this review likely reflects this selective reporting and should therefore be interpreted as an indicator of potential clinical severity rather than true population prevalence.

Second, data heterogeneity and incomplete reporting limited the consistency of several variables. Our cohort included formal case reports as well as clinical images, letters, and conference abstracts, many of which lacked granular clinical or procedural details. To address this variability, a modified JBI quality appraisal tool was applied to provide a transparent framework for evaluating reporting quality. Although this approach cannot compensate for missing information, it allows readers to interpret the findings within the context of each report’s methodological completeness.

Third, the absence of standardized reporting protocol across specialties, particularly among spine surgery, radiology, cardiology, and cardiothoracic surgery, contributed to variability in the level of anatomical and procedural detail. Reports authored outside surgical disciplines often provided limited descriptions of intraoperative findings or retrieval maneuvers, reflecting differences in documentation priorities across fields. This variability may have led to underreporting or inconsistent characterization of less dramatic presentations. Despite these constraints, this review offers the most comprehensive synthesis to date of the anatomical and procedural considerations relevant to intracardiac BCE.

## Conclusion

Intracardiac BCE represents a mechanically hazardous condition that can closely mimic acute coronary syndromes. Across 116 published cases, these rigid fragments were frequently associated with cardiac perforation (46.6%) and valvular injury, underscoring the importance of timely recognition. Although transcatheter techniques continue to evolve, current evidence indicates that the risk of fragmentation and the need for definitive structural repair often lead clinicians to select surgical retrieval in more complex presentations.

A heightened index of suspicion is warranted following any confirmed cement leakage during vertebral augmentation, particularly when new cardiopulmonary symptoms arise. Echocardiography or CT can help clarify fragment morphology, mobility, and chamber involvement, which in turn may indicate ongoing mechanical interaction with cardiac structures. Ultimately, vigilance for iatrogenic cement migration and careful assessment of fragment characteristics are essential for preventing progressive injury from these “intracardiac spears.”

## Electronic Supplementary Material

Supplementary Material 1

## Data Availability

The authors confirm that the data are available from the corresponding author upon reasonable request.

## Abbreviations

ACS: Acute coronary syndrome
ASD: Atrial septal defect
BCE: Bone cement embolism
CPB: Cardiopulmonary bypass
CT: Computed tomography
IVC: Inferior vena cava
JBI: Joanna Briggs Institute
LA: Left atrium
LV: Left ventricle
PFO: Patent foramen ovale
PMMA: Polymethylmethacrylate
PRISMA: Preferred Reporting Items for Systematic Reviews and Meta-Analyses
RA: Right atrium
RV: Right ventricle
SVC: Superior vena cava
