# Supplementary material for: Intracardiac bone cement embolism and cardiac injury: a systematic review of 116 reported cases
Source: J Cardiothorac Surg. 2026 Jun 20;21:693. doi: 10.1186/s13019-026-04453-7 (PMC13563703; doi:10.1186/s13019-026-04453-7)
Supplement: Supplementary file 1 [file 13019_2026_4453_MOESM1_ESM.docx]

**Supplementary Appendix**

**Appendix 1. Search Strategies for Each Database**

**PubMed (via the National Library of Medicine)**

{"Bone Cements"[Mesh] OR "Methylmethacrylate"[Mesh] OR "Bone Cement*"[TW] OR "Bone Glue*"[TW] OR "Bone Paste*"[TW]} AND {"Heart"[Mesh] OR "Venae Cavae"[Mesh] OR "Pulmonary Artery"[Mesh] OR "Pulmonary Veins"[Mesh] OR "Aorta"[Mesh] OR "Heart*"[TW] OR "Cardi*"[TW] OR "Ventric*"[TW] OR "Atri*"[TW] OR "Valv*"[TW] OR "Mitral*"[TW] OR "Tricuspid*"[TW] OR "Pulmon*"[TW] OR "Pericardi*"[TW] OR "Superior Vena Cava"[TW] OR "Inferior Vena Cava"[TW] OR "Aort*"[TW] OR "Cardiovascular*"[TW] OR "Intracardi*"[TW]} AND {"Heart Injuries"[Mesh] OR "Heart Diseases"[Mesh] OR "Rupture"[Mesh] OR "Vascular System Injuries"[Mesh] OR "Cardiovascular Surgical Procedures"[Mesh] OR "Embolism and Thrombosis"[Mesh] OR "Foreign-Body Migration"[Mesh] OR "Injur*"[TW] OR "Rupture*"[TW] OR "Tear*"[TW] OR "Disrupt*"[TW] OR "Tamponade*"[TW] OR "Effusion*"[TW] OR "Hemopericardi*"[TW] OR "Perforat*"[TW] OR "Surg*"[TW] OR "Procedur*"[TW] OR "Endovascular*"[TW] OR "Embol*"[TW] OR "Leak*"[TW] OR "Foreign Bod*"[TW] OR "Foreign-Bod*"[TW] OR "Migration*"[TW] OR "Insufficienc*"[TW] OR "Incompetenc*"[TW] OR "Regurgita*"[TW]}

**Embase (via Elsevier)**

{'bone cement'/exp OR 'methacrylic acid methyl ester'/exp OR 'Bone Cement*':ab,ti,kw OR 'Bone Glue*':ab,ti,kw OR 'Bone Paste*':ab,ti,kw} AND {'heart'/exp OR 'cava vein'/exp OR 'pulmonary artery'/exp OR 'pulmonary vein'/exp OR 'aorta'/exp OR 'Heart*':ab,ti,kw OR 'Cardi*':ab,ti,kw OR 'Ventric*':ab,ti,kw OR 'Atri*':ab,ti,kw OR 'Valv*':ab,ti,kw OR 'Mitral*':ab,ti,kw OR 'Tricuspid*':ab,ti,kw OR 'Pulmon*':ab,ti,kw OR 'Pericardi*':ab,ti,kw OR 'Intracardi*':ab,ti,kw OR 'Superior Vena Cava':ab,ti,kw OR 'Inferior Vena Cava':ab,ti,kw OR 'Aort*':ab,ti,kw OR 'Cardiovascular*':ab,ti,kw} AND {'heart disease'/exp OR 'rupture'/exp OR 'blood vessel injury'/exp OR 'cardiovascular surgery'/exp OR 'embolism'/exp OR 'foreign body'/exp OR 'Injur*':ab,ti,kw OR 'Rupture*':ab,ti,kw OR 'Tear*':ab,ti,kw OR 'Disrupt*':ab,ti,kw OR 'Tamponade*':ab,ti,kw OR 'Effusion*':ab,ti,kw OR 'Hemopericardi*':ab,ti,kw OR 'Perforat*':ab,ti,kw OR 'Surg*':ab,ti,kw OR 'Procedur*':ab,ti,kw OR 'Endovascular*':ab,ti,kw OR 'Embol*':ab,ti,kw OR 'Leak*':ab,ti,kw OR 'Foreign Bod*':ab,ti,kw OR 'Foreign-Bod*':ab,ti,kw OR 'Migration*':ab,ti,kw OR 'Insufficienc*':ab,ti,kw OR 'Incompetenc*':ab,ti,kw OR 'Regurgita*':ab,ti,kw}

**Cochrane Library (via Wiley)**

{[mh "Bone Cements"] OR [mh "Methylmethacrylate"] OR (Bone Cement*):ab,ti,kw OR (Bone Glue*):ab,ti,kw OR (Bone Paste*):ab,ti,kw} AND {[mh "Heart"] OR [mh "Venae Cavae"] OR [mh "Pulmonary Artery"] OR [mh "Pulmonary Veins"] OR [mh "Aorta"] OR Heart*:ab,ti,kw OR Cardi*:ab,ti,kw OR Ventric*:ab,ti,kw OR Atri*:ab,ti,kw OR Valv*:ab,ti,kw OR Mitral*:ab,ti,kw OR Tricuspid*:ab,ti,kw OR Pulmon*:ab,ti,kw OR Pericardi*:ab,ti,kw OR Intracardi*:ab,ti,kw OR (Superior NEXT Vena NEXT Cava):ab,ti,kw OR (Inferior NEXT Vena NEXT Cava):ab,ti,kw OR Aort*:ab,ti,kw OR Cardiovascular*:ab,ti,kw} AND {[mh "Heart Injuries"] OR [mh "Heart Diseases"] OR [mh "Rupture"] OR [mh "Vascular System Injuries"] OR [mh "Cardiovascular Surgical Procedures"] OR [mh "Embolism and Thrombosis"] OR [mh "Foreign-Body Migration"] OR Injur*:ab,ti,kw OR Rupture*:ab,ti,kw OR Tear*:ab,ti,kw OR Disrupt*:ab,ti,kw OR Tamponade*:ab,ti,kw OR Effusion*:ab,ti,kw OR Hemopericardi*:ab,ti,kw OR Perforat*:ab,ti,kw OR Surg*:ab,ti,kw OR Procedur*:ab,ti,kw OR Endovascular*:ab,ti,kw OR Embol*:ab,ti,kw OR Leak*:ab,ti,kw OR (Foreign NEXT Bod*):ab,ti,kw OR Foreign-Bod*:ab,ti,kw OR Migration*:ab,ti,kw OR Insufficienc*:ab,ti,kw OR Incompetenc*:ab,ti,kw OR Regurgita*:ab,ti,kw}

**Appendix 2. Operational Definitions for Quality Appraisal (Modified JBI Tool)**

The reporting quality of the included studies was assessed using a modified Joanna Briggs Institute l(JBI) critical appraisal checklist adapted for the specific clinical context of intracardiac bone cement embolism.

| Item | Domain | Score 1.0  (Complete) | Score 0.5  (Partial) | Score 0.0  (Incomplete) |
| --- | --- | --- | --- | --- |
| Q1 | Demographics | Both [age] and [sex] are explicitly reported. | Only one of [age] or [sex] is reported. | Neither age nor sex is reported. |
| Q2 | History/exposure | Spinal procedures:  Specific level is stated.  Non-spinal: The primary procedure is identified. | The spinal procedure is mentioned, but the specific level is missing. | The type of the primary procedure is not mentioned. |
| Q3 | Clinical condition | Initial symptoms are described, or the “asymptomatic” status is stated. | - | No mention of the initial clinical status. |
| Q4 | Modality/timeline | The diagnostic modality is identified, AND the interval from procedure is stated. | Modality is identified, but timing/interval is missing. | Both modality and timing information are missing. |
| Q5 | Management | 1) Conservative/endovascular: The strategy is explicitly stated.  2) Surgery: Both surgical approach AND CPB status are explicitly reported. | Surgery only: Only surgical intervention is mentioned, without reporting both approach and CPB status. | No mention of the management strategy or treatment type. |
| Q6 | Embolus morphology | Both [shape] and [size] are explicitly described. | Only one of [shape] or [size] is described. | Neither shape nor size is mentioned. |
| Q7 | Clinical outcome | Clear description of the discharge status or postprocedural outcome. | Brief or vague mention of the clinical status. | No follow-up or outcome status was reported. |
| **Q8 | Take-away message | At least one clear clinical lesson or practice implication is provided. | - | No take-away message was provided. |

**Appendix 3. Inter-rater Reliability (Fleiss’ Kappa) by Domain**

This table summarizes the agreement among the three independent cardiac surgeons (R1, R2, and R3) for each appraisal domain before consensus resolution.

| **Appraisal domain** | **Fleiss’ kappa (κ)** | **Strength of agreement^*^** | **Mean consensus score**^†^ |
| --- | --- | --- | --- |
| **Q1. Demographics** | 0.87 | Almost perfect | 98.7% |
| **Q2. History/exposure** | 0.93 | Almost perfect | 92.7% |
| **Q3. Clinical presentation** | 1.00 | Almost perfect | 99.1% |
| **Q4. Diagnostic information** | 0.90 | Almost perfect | 98.3% |
| **Q5. Management strategy** | 0.89 | Almost perfect | 90.1% |
| **Q6. Embolus morphology** | 0.71 | Substantial | 87.9% |
| **Q7. Outcomes** | 0.80 | Substantial | 93.1% |
| **Q8. Take-away message** | 0.30 | Fair | 86.2% |
| **Overall (all domains)** | 0.77 | Substantial | 93.3% |

^*^Interpretation based on Landis and Koch (1977): <0.00, poor; 0.01–0.20, slight; 0.21–0.40, fair; 0.41–0.60, moderate; 0.61–0.80, substantial; 0.81–1.00, almost perfect.

^†^The mean consensus score represents the average reporting completeness across 116 cases, where 100% indicates full documentation in all categories.

**Appendix 4. References of Included Cases**

**S1.** Zhang X, Zhang H, Li Z. Cement embolism in the right ventricle. *European Heart Journal Cardiovascular Imaging.* 2025;26:1602.

**S2.** Pu J, He JQ, Yi Q, Zhou H. Simultaneous pulmonary and intracardiac bone cement embolism after vertebroplasty: a case report and literature review. *J Cardiothorac Surg.* 2025;20:405.

**S3.** Oudih M, Al-Taei M, Oudih M, Jahufar F, Al-Abduladheem A, Patel S. A Mysterious Piece Of Cement In The Heart; Diagnosis And Management Of Intracardiac Bone Cement Embolism*.* Vol 312025:215.

**S4.** Liu R, Wang Z, Zhang Z, et al. A rare case of right atrial perforation and pericardial tamponade following leakage of bone cement. *J Cardiothorac Surg.* 2025;20:174.

**S5.** Kong D, Xie X, Jin Y, Zhang Y. Case Report: Bone cement leakage in the right heart: a rare case of misinterpreted echocardiographic findings. *Front Surg.* 2025;12:1608784.

**S6.** Dai X, Chen L, Pan D, Zhao H, Ma L. Bone cement embolism causing right ventricle perforation. *BMC Cardiovasc Disord.* 2025;25:101.

**S7.** Chen F, Li C, Chen YJ, Hu YZ. Diagnosis of isolated intracardiac cement embolism causing cardiac rupture via bedside transthoracic echocardiography: a case report. *BMC Cardiovasc Disord.* 2025;25:574.

**S8.** Zhao Z, Wang R, Gao L, Zhang M. Pulmonary embolism and intracardiac foreign bodies caused by bone cement leakage: a case report and literature review. *J Cardiothorac Surg.* 2024;19:544.

**S9.** Yang Y, Fei Q, Long GQ, et al. Cardiac cement embolism and asymptomatic pulmonary embolism caused by percutaneous vertebroplasty for osteoporotic vertebral fracture: a case report. *Front Surg.* 2024;11:1464049.

**S10.** Wu SX, Huang C, Xu J. Perforation of the right ventricle and pulmonary artery embolism by bone cement after percutaneous vertebroplasty. *Eur Heart J.* 2024;45:2680.

**S11.** Wang J, Huang Y, Liu ZM, Zeng H. [A case report of massive pericardial effusion caused by bone cement treatment]. *Zhonghua Xin Xue Guan Bing Za Zhi.* 2024;52:929–931.

**S12.** Mastroiacovo G, Leka E, Marchetti F, Maragna R, Carulli E, Agrifoglio M. A Case Report Describing the Surgical Removal of Venous and Intracardiac Cement Leakage after Percutaneous Vertebroplasty in a Hybrid Operating Room. *Ann Ital Chir.* 2024;95:1072–1075.

**S13.** Liu Y, Gong L, Yang M. Symptomatic pulmonary and intracardiac cement embolism after percutaneous vertebroplasty: A case report. *Asian J Surg.* 2024.

**S14.** Li J, Zhang Y, Zhang H, Jiang S, Wu X. Intracardiac leakage of cement during artificial femoral head replacement: a case report and review. *Int J Clin Exp Pathol.* 2024;17:72–77.

**S15.** Ghoweba M, Rao P, Chiluveri M, McClish J. A BACKSTAB TO THE HEART - KYPHOPLASTY CEMENT PERFORATING THE RIGHT ATRIUM. *Journal of the American College of Cardiology.* 2024;83:3863.

**S16.** Feng X, Zhou Q. Cement arrow in the heart: an unusual vertebroplasty complication. *Eur Heart J.* 2024;45:2265.

**S17.** Choi KU, Park JI, Nam JH, Lee CH, Son JW. Sharp foreign body causing haemopericardium: bone cement fragment. *Eur Heart J Cardiovasc Imaging.* 2024;25:e152.

**S18.** Wei Z, Weng X. Intracardiac and intrapulmonary cement embolisms after percutaneous vertebroplasty. *Quantitative Imaging in Medicine and Surgery.* 2023;13:8850–8852.

**S19.** Waidyaratne G, Bennett C, Umyarova E, Bumma N. EXTENSIVE INTRA CAR DIAC CEMENT EMBOLISM IN A PATIENT UNDERGOING WORKUP FOR BONE MARROW TRANSPLANT. *Journal of General Internal Medicine.* 2023;38:S587.

**S20.** Tseng CC, Chang PC, Chang TW. Cement embolism in the right ventricle. *Kaohsiung J Med Sci.* 2023;39:193–194.

**S21.** Schmiady MO, Possner M, Horisberger T, Ouda A. Intracardiac bone cement embolism resulting in ventricular perforation: an unusual cause of sudden chest pain. *Interdiscip Cardiovasc Thorac Surg.* 2023;36.

**S22.** Peng Y, Xia R, Zhao D. Bone cement migrated to the right heart resulting in perforation of the ventricular wall with cardiac tamponade. *Eur Heart J.* 2023;44:2133.

**S23.** Liu X, Chu P, Miao Q. Case report: Open-heart removal for a cement embolism formed 10 years ago in the right ventricle and pulmonary artery. *Front Cardiovasc Med.* 2023;10:1221525.

**S24.** Jain A, Al-Bermani T, Shah DJ, Epelbaum O. A Case of Cardiac and Pulmonary Cement Embolism (CPCE) After Percutaneous Vertebroplasty. *American Journal of Respiratory and Critical Care Medicine.* 2023;207.

**S25.** Deng K, Yu JL, Feng YJ, Huang K, Wu GF. Case report: Inspiration from a rare fatal heart perforation after percutaneous vertebroplasty. *Front Surg.* 2023;10:1227056.

**S26.** Tang G, Zhang M, Wu P, Chen Y-A, Hou J-J, Zhang J. Bone cement ectopic embolism of right ventricular complicated with cardiac tamponade after percutaneous vertebroplasty: Case report. *Chinese Journal of Interventional Imaging and Therapy.* 2023;20:595.

**S27.** Zhang Y, Liu X, Liu H. Cardiac Perforation Caused by Cement Embolism after Percutaneous Vertebroplasty: A Report of Two Cases. *Orthop Surg.* 2022;14:456–460.

**S28.** Yang KJ, Lee YP, Huang CH, Wang CH. Bone cement embolism into the right heart. *JTCVS Tech.* 2022;16:64–67.

**S29.** Trongtorsak A, Saad E, Mustafa A, et al. Incidental detection of an intracardiac cement embolism complicating percutaneous vertebroplasty during cardiac catheterization: A case report. *J Cardiol Cases.* 2022;26:151–153.

**S30.** Sun K, Huang F, Liang B. A case report of intracardiac bone cement embolization after posterior decompression and cement-enhanced pedicle screw fixation for osteoporosis and lumbar degeneration. *Medicine (Baltimore).* 2022;101:e28826.

**S31.** Shin CS, Kim MW, Go JH, Lee JM, Choi JB. Bone cement embolism penetrating both the aorta and the right atrium after percutaneous vertebroplasty: Thermal rather than mechanical injury? *JTCVS Tech.* 2022;14:43–44.

**S32.** Mills S, Pizones J, Merino Rueda LR, Talavera G, Sánchez-Márquez JM, Fernández-Baíllo N. Cardiac Cement Embolism After Thoracic Kyphoplasty: Successful Conservative Treatment With 4-Year Follow-Up. *Int J Spine Surg.* 2022;16:27–32.

**S33.** Lee JY, Lee SH, Kim WH. Migrated Bone Cement Impending Cardiac Perforation. *Anatol J Cardiol.* 2022;26:5004–5005.

**S34.** Krithika R, Geske JB, Villarraga HR, Crestanello J. Concrete proof of Murphy’s law: a case report of intracardiac cement embolization. *European Heart Journal - Case Reports.* 2022;6.

**S35.** Huang C. Life-threatening intracardiac cement embolisms after percutaneous kyphoplasty: a case report and literature review. *J Int Med Res.* 2022;50:3000605221102088.

**S36.** Byoun JT, Lee SY, Cho JY, Yun KH, Oh SK. Hemopericardium with Cardiac Tamponade after Percutaneous Vertebroplasty. *Korean Circulation Journal.* 2022;52.

**S37.** Bassawon R, Sirajuddin S, Martucci G, Shum-Tim D. Snare or Scalpel: Challenges of Intracardiac Cement Embolism Retrieval. *Ann Thorac Surg.* 2022;113:e107-e110.

**S38.** Bale BA, Mishra C, Kabiria B. CEMENTING THE SIDE EFFECTS OF KYPHOPLASTY: CEMENT EMBOLI IN THE CARDIOPULMONARY SYSTEM. *Chest.* 2022;162:A2405.

**S39.** Yoon JC, Kim SE. Chest Pain From an Unusual Cause: Intracardiac Leaked Bone Cement After Percutaneous Vertebroplasty. *J Emerg Med.* 2021;61:e120-e122.

**S40.** Yin P, Hu J, Wang S, Sui G, Yuan G, Fan D. Bedside echocardiography for diagnosis of intracardiac cement embolism after percutaneous vertebroplasty: A case report. *J Card Surg.* 2021;36:3929–3932.

**S41.** Weininger G, Sekar RB, Elefteriades JA. Intracardiac polymethylmethacrylate cement embolism. *JTCVS Techniques.* 2021;10:358–360.

**S42.** Weininger G, Elefteriades JA. Intracardiac Cement Embolism. *N Engl J Med.* 2021;385:e49.

**S43.** Wang B, Li Y, Peng Y, et al. Vertebroplasty and right heart cement embolism. *Qjm.* 2021;114:124–126.

**S44.** Van De Ree-Pellikaan C, Schouwenberg PHM, Ter Horst M, Den Uil CA. Right atrial perforation from intracardiac cement embolus after vertebroplasty. *European Heart Journal - Case Reports.* 2021;5.

**S45.** Takahashi S, Sohgawa E, Suzuki A, et al. Percutaneous endovascular retrieval of large cement leakage into the right atrium following cement-augmented pedicle screw instrumentation. *Spine Surgery and Related Research.* 2021;5:446–449.

**S46.** Qiao C, Lv M, Wang Y. Intracardial Cement in Postanesthesia Care Unit. *Anesthesiology.* 2021;135:893.

**S47.** Pan HY, Wu TY, Chen HY, Wu JY, Liao MT. Intracardiac Cement Embolism: Images and Endovascular Treatment. *Circ Cardiovasc Imaging.* 2021;14:e011849.

**S48.** Mathy RM, Kuhn TC, Kappes J, et al. Intracardial PMMA bone cement embolism after kyphoplasty-an unusual cause for sudden chest pain, hemothorax and hemopericardium. *Med Klin Intensivmed Notfmed.* 2021;116:61–64.

**S49.** Lee S, Yeon JW, Kwon JT, Kim HJ, Jang SK. Transcatheter Removal of Bone Cement Embolism in the Right Atrium after Percutaneous Vertebroplasty: The Embolus Broke in Half and Migrated to the Right Pulmonary Artery Intraoperatively. *Taehan Yongsang Uihakhoe Chi.* 2021;82:1011–1017.

**S50.** Kim SJ, Kim KH. Unusual Pneumothorax with Cardiac Perforation by Bone Cement after Percutaneous Vertebroplasty. *Am J Respir Crit Care Med.* 2021;203:e29-e30.

**S51.** Armashi AA, Somoza-Cano F, Farra Y. A SLEEPING SPEAR WITHIN THE HEART, A CATASTROPHE IN LOOM. *Journal of the American College of Cardiology.* 2021;77:3014.

**S52.** Song Y, Huang X, Wu L. Removal of intracardiac bone cement embolism after percutaneous kyphoplasty: A case report. *Medicine (Baltimore).* 2020;99:e19354.

**S53.** Shamekhi J, Düsing P, Sedaghat A, Kütting D, Nickenig G, Sinning JM. Healing a Heart of Stone: Percutaneous Extraction of Cardiopulmonary Cement Embolism. *JACC Cardiovasc Interv.* 2020;13:532–533.

**S54.** Plateker O, Daix T, Boulogne C, Rousselle V, Vignon P, Porterie J. Post-kyphoplasty cement embolism migrating to the peritoneum through the right ventricle. *J Cardiovasc Comput Tomogr.* 2020;14:e159-e160.

**S55.** Hong SW, Oh TH, Jeon JM, Lee YS, Kim KT. Cardiac Perforation Caused by Bone Cements as a Complication of Cement Augmented Pedicle Screw Fixation Using the Fenestrated Pedicle Screw: A Case Report. *Korean J Neurotrauma.* 2020;16:337–342.

**S56.** Akhunova S, Khayrullin R, Stekolshchikova N, Samigullin M, Padiryakov V. Right atrial, right ventricular and pulmonary cement embolism: A rare complication of percutaneous vertebroplasty. *European Heart Journal Cardiovascular Imaging.* 2020;21:i385.

**S57.** Teng F, Xu XJ, Liu Q. A modified wire-loop snare technique for the retrieval of a large cardiac cement embolus caused by cement leakage after percutaneous vertebroplasty. *Journal of Interventional Medicine.* 2019;2:38–41.

**S58.** Kong M, Xu X, Shen J, Liu Q, Wang G. Clinical characteristics and management of cardiac and/or pulmonary cement embolus after percutaneous vertebroplasty: a single center experience. *Ann Transl Med.* 2019;7:372.

**S59.** Duijvelshoff R, Anthonissen NFM, Morshuis WJ, Van Garsse L. Intracardiac cement embolism resulting in tricuspid regurgitation. *Eur J Cardiothorac Surg.* 2019;55:366–368.

**S60.** D'Errico S, Niballi S, Bonuccelli D. Fatal cardiac perforation and pulmonary embolism of leaked cement after percutaneous vertebroplasty. *J Forensic Leg Med.* 2019;63:48–51.

**S61.** Craba L, Centorbi C, Zanda G, et al. Echocardiographic accidental finding of asymptomatic cardiac and pulmonary embolism due to cemental leakeage after percutaneous vertebroplasty. *Giornale Italiano di Cardiologia.* 2019;20:128S-129S.

**S62.** Bauer M, Kröger U, Lenga P. [Dyspnoea Due to Foreign Embolism after Percutaneous Vertebroplasty]. *Dtsch Med Wochenschr.* 2019;144:1069–1073.

**S63.** Alekhin MN, Ter-Akopian AV, Abramov AS, Skripnikova AV. [Intracardiac and Pulmonary Cement Embolism after Percutaneous Vertebroplasty]. *Kardiologiia.* 2019;59:92–96.

**S64.** Adu-Gyamfi KO, Patri S. Symptomatic cardiopulmonary cement embolism following vertebroplasty. *BMJ Case Rep.* 2019;12.

**S65.** Swojanowsky P, Brinkmeier-Theofanopoulou M, Schmitt C, Mehlhorn U. A rare cause of pericardial effusion due to intracardiac cement embolism. *Eur Heart J.* 2018;39:3001.

**S66.** Rott C, Roithinger FX, Gremmel T. Bone cement in the right heart. *Eur Heart J Cardiovasc Imaging.* 2018;19:825.

**S67.** Park JS, Kim J, Lee Y, Gwon JG, Park YS. Intra-cardiac Embolism of a Large Bone Cement Material after Percutaneous Vertebroplasty Removed through a Combination of an Endovascular Procedure and an Inferior Vena Cava Exploration: a Case Report. *J Korean Med Sci.* 2018;33:e141.

**S68.** Oshinsky C, Bhavani S, Funaki A. Cement cardiac embolism following kyphoplasty noted on thoracic imaging. *Radiology Case Reports.* 2018;13:914–916.

**S69.** Elens M, Lecouvet F. Vertebral Cement Leak: How Far Will It Go…? *Eur J Vasc Endovasc Surg.* 2018;55:416.

**S70.** Barakat AS, Owais T, Alhashash M, et al. Presentation and management of symptomatic central bone cement embolization. *Eur Spine J.* 2018;27:2584–2592.

**S71.** Ishikawa K, Hayashi H, Mori H. Cardiac perforation caused by cement after percutaneous balloon kyphoplasty. *Asian Cardiovasc Thorac Ann.* 2017;25:204–206.

**S72.** Hatzantonis C, Czyz M, Pyzik R, Boszczyk BM. Intracardiac bone cement embolism as a complication of vertebroplasty: management strategy. *Eur Spine J.* 2017;26:3199–3205.

**S73.** Cianciulli TF, Mc Loughlin DE, Morita LA, Saccheri MC, Lax JA. Bone cement cardiac and pulmonary embolism. *Echocardiography.* 2017;34:1239–1241.

**S74.** Andrä M, Baumer H, Mittergradnegger F, Laschitz M, Petek T, Wandschneider W. Life-Threatening Cardiac Perforation After Posterior Spondylodesis. *Ann Thorac Surg.* 2017;104:e355-e357.

**S75.** Shridhar P, Chen Y, Khalil R, et al. A Review of PMMA Bone Cement and Intra-Cardiac Embolism. *Materials (Basel).* 2016;9.

**S76.** Molloy T, Kos A, Piwowarski A. Robotic-Assisted Removal of Intracardiac Cement After Percutaneous Vertebroplasty. *Ann Thorac Surg.* 2016;101:1974–1976.

**S77.** Kim IC, Hong GR, Shim CY, Lee S. A trace of acrylic bone cement: from spine to heart. *Eur Heart J Cardiovasc Imaging.* 2016;17:470.

**S78.** Elapavaluru S, Alhassan S, Khan F, Khalil R, Schuett A, Bailey S. Severe acute traumatic mitral regurgitation, cardiogenic shock secondary to embolized polymethylmethracrylate cement foreign body after a percutaneous vertebroplasty. *Annals of Thoracic Surgery.* 2016;101:1169–1171.

**S79.** Diab MS, Diab A, Dihmis W, Diab S. Acute right atrial and pulmonary artery bone cement mass emboli following vertebroplasty. *JRSM Open.* 2016;7:2054270416643891.

**S80.** Audat ZA, Alfawareh MD, Darwish FT, Alomari AA. Intracardiac Leakage of Cement During Kyphoplasty and Vertebroplasty: A Case Report. *Am J Case Rep.* 2016;17:326–330.

**S81.** Von Ballmoos MW, Almassi G, Gandhi S. Emergent embolectomy for massive intraoperative thromboembolism during total artificial hip replacement. *Chest.* 2015;148.

**S82.** Skjølsvik ET, Steensrud T, Dahl-Eriksen Ø, Uhlin-Hansen L, Lunde PI. Cardiac Perforation Caused by Cement Embolus After Total Hip Replacement. *Circulation.* 2015;132:1047–1048.

**S83.** Shen C, Liu G, Hu JZ, Yang XH. Cardiac Perforation and Multiple Emboli After Percutaneous Vertebroplasty. *Orthopedics.* 2015;38:e947-950.

**S84.** Schulze B, Hoffmann R, Scholtz W, Gummert J, Paluszkiewicz L. Uncommon intracardiac finding after cardiac transplantation mimicking pacemaker leads. *Eur Heart J Cardiovasc Imaging.* 2015;16:571.

**S85.** Schuerer S, Misfeld M, Schuler G, Mangner N. Intracardiac cement embolization in a 65-year-old man four months after multilevel spine fusion. *Eur Heart J.* 2015;36:783.

**S86.** Polli FM, Marongiu A, Miscusi M, De-Giorgio F, Raco A. Cardiopulmonary cement embolism after vertebroplasty. *Spine J.* 2015;15:376.

**S87.** Oh JK, Park JH, Kim SS, et al. Thread-like Bone Cement in the Right-side Heart and Pulmonary Arteries Causing Diffuse Pulmonary Embolism as a Late Complication. *Heart Lung Circ.* 2015;24:e104-107.

**S88.** George KM, Campbell M. Embolized methyl methacrylate to the right atrium after kyphoplasty. *Ann Thorac Surg.* 2015;99:337.

**S89.** Wiedemann S, Ebner B, Ibrahim K, Scherf L, Heidrich FM, Strasser RH. Percutaneous transjugular removal of an intracardial bone cement fragment after dorsal stabilization. *Circ Cardiovasc Interv.* 2014;7:410–413.

**S90.** Pannirselvam V, Hee HT. Asymptomatic cement embolism in the right atrium after vertebroplasty using high-viscosity cement: a case report. *J Orthop Surg (Hong Kong).* 2014;22:244–247.

**S91.** Lee V, Patel R, Meier P, Lawrence D, Roberts N. Conservative management of inferior vena cava cement spike after percutaneous vertebroplasty causes fatal cardiac tamponade. *J Rheumatol.* 2014;41:141–142.

**S92.** Kim SP, Son BS, Lee SK, Kim DH. Cardiac perforation due to intracardiac bone cement after percutaneous vertebroplasty. *J Card Surg.* 2014;29:499–500.

**S93.** Berthoud B, Sarre G, Chaix D, Ennezat PV. Cardiac tamponnade, cement right atrial mass, and pulmonary embolism complicating percutaneous plasty of osteolytic metastases. *Eur Heart J.* 2014;35:2333.

**S94.** Yang JH, Kim JW, Park HO, Choi JY, Jang IS, Lee CE. Intracardiac foreign body (bone cement) after percutaneous vertebroplasty. *Korean J Thorac Cardiovasc Surg.* 2013;46:72–75.

**S95.** Tran I, Gerckens U, Remig J, Zintl G, Textor J. First report of a life-threatening cardiac complication after percutaneous balloon kyphoplasty. *Spine (Phila Pa 1976).* 2013;38:E316-318.

**S96.** Prokop A, Hägele M, Pfeilsticker U, Koll S, Chmielnicki M. [Pericardial perforation 2.5 years after kyphoplasty. A rare complication after cement extravasation]. *Unfallchirurg.* 2013;116:80–84.

**S97.** Moon MH, Jo KH, Kim HW. Cardiac perforation caused by bone cement embolism. *Arch Cardiovasc Dis.* 2013;106:413–414.

**S98.** Llanos RA, Viana-Tejedor A, Abella HR, Fernandez-Avilés F. Pulmonary and intracardiac cement embolism after a percutaneous vertebroplasty. *Clin Res Cardiol.* 2013;102:395–397.

**S99.** Kim HT, Kim YN, Shin HW, et al. Intracardiac foreign body caused by cement leakage as a late complication of percutaneous vertebroplasty. *Korean J Intern Med.* 2013;28:247–250.

**S100.** Grifka RG, Tapio J, Lee KJ. Transcatheter retrieval of an embolized methylmethacrylate glue fragment adherent to the right atrium using bidirectional snares. *Catheter Cardiovasc Interv.* 2013;81:648–650.

**S101.** Gosev I, Nascimben L, Huang PH, et al. Right ventricular perforation and pulmonary embolism with polymethylmethacrylate cement after percutaneous kyphoplasty. *Circulation.* 2013;127:1251–1253.

**S102.** Dreger H, Treskatsch S, Lembcke A, Grubitzsch H, Knebel F, Laule M. Perforation of the right ventricle by bone cement: a rare complication of kyphoplasty. *Eur Heart J.* 2013;34:1203.

**S103.** Szolnoky J, Leber A, Kotetishvili N, Eszlari E, Gansera B, Eichinger W. Surgical removal of intracardiac-intrapulmorary orthopedic cement embolism after percutaneous vertebroplasty. *Thoracic and Cardiovascular Surgeon.* 2012;60.

**S104.** Mattis T, Knox M, Mammen L. Intracardiac methylmethacrylate embolism resulting in right atrial wall perforation and pericarditis following percutaneous vertebroplasty. *J Vasc Interv Radiol.* 2012;23:719–720.

**S105.** Czesla M, Karnari O, Götte J, et al. Unusual cause of acute back pain mimicking aortic dissection: a case report. *Thorac Cardiovasc Surg.* 2012;60 Suppl 2:e28-30.

**S106.** Cohen JB. Bone cement embolism. *Anesthesiology.* 2012;117:407.

**S107.** Chung JW, Shin JK, Chee HK, Kim JS, Kim DC, Park JB. Removal of Bone Cement through Right Anterolateral Thoracotomy. *Korean J Thorac Cardiovasc Surg.* 2012;45:202–204.

**S108.** Dash A, Brinster DR. Open heart surgery for removal of polymethylmethacrylate after percutaneous vertebroplasty. *Ann Thorac Surg.* 2011;91:276–278.

**S109.** Lee JS, Jeong YS, Ahn SG. Intracardiac bone cement embolism. *Heart.* 2010;96:387.

**S110.** Kim MN, Jung JS, Kim SW, Kim YH, Park SM, Shim WJ. A sword-like foreign body lodged in the ventricular septum: a rare complication of percutaneous vertebroplasty. *Eur Heart J.* 2010;31:1006.

**S111.** Farahvar A, Dubensky D, Bakos R. Perforation of the right cardiac ventricular wall by polymethylmethacrylate after lumbar kyphoplasty. *J Neurosurg Spine.* 2009;11:487–491.

**S112.** Cadeddu C, Nocco S, Secci E, Deidda M, Pirisi R, Mercuro G. Echocardiographic accidental finding of asymptomatic cardiac and pulmonary embolism caused by cement leakage after percutaneous vertebroplasty. *Eur J Echocardiogr.* 2009;10:590–592.

**S113.** Braiteh F, Row M. Right ventricular acrylic cement embolism: Late complication of percutaneous vertebroplasty. *Heart.* 2009;95:275.

**S114.** Son KH, Chung JH, Sun K, Son HS. Cardiac perforation and tricuspid regurgitation as a complication of percutaneous vertebroplasty. *Eur J Cardiothorac Surg.* 2008;33:508–509.

**S115.** Schoenes B, Bremerich DH, Risteski PS, Thalhammer A, Meininger D. [Cardiac perforation after vertebroplasty]. *Anaesthesist.* 2008;57:147–150.

**S116.** Lim SH, Kim H, Kim HK, Baek MJ. Multiple cardiac perforations and pulmonary embolism caused by cement leakage after percutaneous vertebroplasty. *Eur J Cardiothorac Surg.* 2008;33:510–512.

**S117.** Lim KJ, Yoon SZ, Jeon YS, et al. An intraatrial thrombus and pulmonary thromboembolism as a late complication of percutaneous vertebroplasty. *Anesth Analg.* 2007;104:924–926.

**S118.** Sang EL, Chang SA, Kim MS, et al. Acrylic cement foreign body and thrombus in right atrium causing pulmonary embolism after percutaneous vertebroplasty. *Korean Circulation Journal.* 2006;36:713–715.

**S119.** Park JH, Choo SJ, Park SW. Images in cardiovascular medicine. Acute pericarditis caused by acrylic bone cement after percutaneous vertebroplasty. *Circulation.* 2005;111:e98.

**S120.** Kim SY, Seo JB, Do KH, Lee JS, Song KS, Lim TH. Cardiac perforation caused by acrylic cement: a rare complication of percutaneous vertebroplasty. *AJR Am J Roentgenol.* 2005;185:1245–1247.
